# Enhancement of Colour Effects of Dyed-Yarn Mixed Fabrics Using Cramming Motion and Finer Polyester Yarns

**DOI:** 10.3390/polym10070783

**Published:** 2018-07-16

**Authors:** Lau Yiu Tang, Xiao Tian, Tao Hua

**Affiliations:** Institute of Textiles and Clothing, The Hong Kong Polytechnic University, Hung Hom, Kowloon, Hong Kong, China; lauyiu.tang@connect.polyu.hk (L.Y.T.); xiao.tian@polyu.edu.hk (X.T.)

**Keywords:** color performance, fabric structure, cramming motion, geometrical parameters, physical properties

## Abstract

This paper reports the study of the effects of cramming motion implemented during weaving and finer weft yarns used on dyed-yarn mixed woven fabrics produced by using raw white warps and multicolored-wefts. The cramming motion was used to increase the dyed-weft yarns cover factor of fabric, and thus, to reduce the negative effect of white warp floats at the fabric face on the color attributes of fabric. The surface structure of fabric was characterized by using several key geometrical parameters that determined the resultant fabric color attributes. The effects of fabric structure and density, weft yarn count, and the introduction of black yarn on the fabric face layer on the fabric surface geometrical parameters, physical properties, as well as color attributes were investigated under the implementation of cramming motion on the fabric. The color attributes of fabrics using cramming motion and finer yarns were also compared to the fabrics without cramming motion. The experimental results indicate that the weft yarn density and cover factor of fabric face layer are increased by applying cramming motion and finer yarns for fabricating the blue-red and/or black mixed fabrics. Consequently, the fabric lightness can be further reduced for achieving a better color effect on colorful and figured woven fabrics mainly using dyed-wefts for color mixing.

## 1. Introduction

Colorful and figured woven fabrics created by using dyed-yarns are characterized by rich color variety, impressive color display, and long-lasting color performance. Due to such characteristics of colorful and figured woven fabrics, this type of fabric has a wide range of applications and outstanding decorative effects. Therefore, they can be commonly found in the environment, such as decorative wall hangings, home decorations, and other products. As colorful and figured woven fabrics are practical and decorative, they are favored by many consumers and are indispensable for the textile industry wherein color is an important element for textiles and always at a prior position within the entire manufacturing process. Individual yarn colors and fabric weaves as well as other structural parameters determine the color performance of the resulting fabrics.

There are a number of past studies on the dyed-yarn based colorful and figured woven fabrics in terms of the selection of warp and weft yarn colors for mixing, fabric structures, effects of fabric structural parameters on fabric color attributes and color prediction models [1,2,3,4,5,6,7]. Eight kinds of dyed wefts, namely white, black, vermillion, yellow, green, blue, Prussian blue and magenta, together with warps in a dark deep green color, were suggested for producing high-quality colorful silk textiles through converting the digital images [8]. Based on the study of the relationship between the color combination of dyed-yarns and color distribution of the resultant color-mixed fabrics, an optimal yarn color combination of red, green, blue, cyan, magenta, and yellow was proposed that can create a relatively full-color gamut for colorful woven fabrics [9]. A double-layer fabric structure under the layered-combination design mode was designed for figured double-face fabric, which can obtain a good covering effect between the face and back layers [10]. Combining with colored yarns used, a compound fabric structure was constructed based on a series of shaded weaves to express colors for the jacquard fabrics [11]. The proportion of color component for mixing and the resulting fabric color performance were determined by the yarn count and density designed for the fabrics [12]. The correction of the color value of woven fabric can be achieved through changing the fabric structure parameters, including the increase of the weft threads density, the change of warp yarn fineness, and the decrease of warp threads density [13]. A method was proposed for calculating the value of color attributes of fabric through a theoretical analysis [14]. The modeling with enhanced predicting precision were suggested for colored-yarn mixed fabrics [15,16,17,18,19]. In order to achieve good color effects of woven fabrics, the main approach for fabricating figured jacquard fabrics is to adopt both multicolored warp and weft yarns. However, the major shortcoming of this approach is the special preparation of warp beams while the weft yarn color can easily change in the weaving process, which limits the flexibility of fabric design and production and lowers the fabric productivity. Therefore, it is meaningful to explore the fabric technology by only using single-colored warps, together with multicolored wefts, for fabricating colorful and figured fabrics. In our previous study, an approach was developed by using white warp threads and colorful weft threads. Two types of specially designed weft-backed structures, one with and the other without the middle regulating layer, were proposed for making use of the colored weft yarns for the color presentation on the fabric face and minimizing the negative effect of white warps on the fabric color attributes, particularly on the fabric lightness. Consequently, a series of dyed-yarn mixed colors were generated that were used for fabricating colorful and figured fabrics [20]. However, it is very necessary to study the relationship between fabric structure and properties for fabric optimization, and further reduce the white warp floats on the fabric surface, thus lowering its negative influence on the desired color effects for achieving better fabric color attributes.

This study aims to enhance the color attributes of the dyed-yarn mixed woven fabrics by (1) reducing the white warp yarn exposed on the fabric face; (2) increasing the face weft density/cover factor of the woven fabric; and (3) fully understanding the relationship between structure and color attributes. In order to achieve the above targets, cramming motion and finer weft yarns were applied for the enhancement of color attributes in colorful and figured woven fabrics. In addition to the color attributes, the impact of structural parameters on physical properties of fabric were also explored so as to optimize the fabric parameters and fabric structure for different applications of the woven fabrics.

## 2. Experimental Details

### 2.1. Fabric Sample Preparation

Based on the designed yarn colors and weft-backed structures in the previous study [20], total forty-four blue-red mixed fabric samples were produced on LX 3202 Staubli Jacquard machine (Staubli, Sargans, Switzerland) for this experiment. Six different colors of red, green, blue, yellow, black, and white were selected for weft yarns, together with white warps and two weft-backed structures; structures with and without middle regulating layers, were used for fabricating the fabric samples. Table 1 lists the detailed fabric specifications of the fabrics produced, and the fabric codes listed here are used in the following sections to express different fabrics.

For the warp yarn, 100 denier (D) polyester filament yarns in white color (We) was applied while for the weft yarn, 75 D and 150 D polyester filament yarns in red (RE), green (GR), blue (BLU), yellow (YE), white (Wp), and black (BLK) were employed in the weaving trials. In order to enhance the color attributes of the fabric, a finer yarn of 75 D polyester filament was used in the fabric production. In addition, the effect of yarn count can also be investigated by comparing the fabric weaved by 150 D yarns with that produced by using 75 D yarns. The yarn color attributes were characterized by using a 7000A spectrophotometer (X-Rite, Incorporated, Grand Rapids, MI, USA). Table 2 lists the tested values of yarn color attributes. The weft-backed structure with the middle regulating layer consists of a face layer; a middle regulating layer, a back layer, and its corresponding weaves are illustrated in Table 3 and Table 4 for blue-red color mixing and blue-red-black color mixing on the fabric face layer, respectively. The weft-backed structure without the middle regulating layer only has a face layer and back layer with detailed weaves for blue-red and blue-red-black color mixings on the fabric top layer shown in Table 5 and Table 6, respectively. Moreover, by varying the mixed yarn color fraction through the weaves from weave M_(BL+R)_ (1) to M_(BL+R)_ (7), N_(BL+R)_ (1) to N_(BL+R)_ (7), M_(BL+R+BK)_ (1) to M_(BL+R+BK)_ (4), and N_(BL+R+BK)_ (1) to N_(BL+R+BK)_ (4), a series of blue–red and blue–red–black color-mixed colors were created on the surface of the fabric. The warp yarn density keeps constant for all fabric samples, which is 47 ends/cm, while the weft yarn density varies with the type of fabric. In order to reduce the negative effect of white warp floats at the fabric face on the color attributes of fabric, particularly on the lightness of the fabric, the function of cramming motion was implemented to lower the spacing between face weft yarns, which means that the weft density of the fabric can be increased. Cramming motion, also called regulator or retarding motion, means that the fabric take-up motion of designate picks is stopped, so that the part of fabric applying cramming motion contains more yarns per unit area [21]. The weft density is 120 picks/cm for the fabrics with and without regulating layer weaved by using 150 denier weft yarns. A lower fabric weft density of 120 picks/cm and a higher weft density of 160 picks/cm were also employed for producing the fabric with the regulating layer by using 75 D weft yarns to examine the effect of weft density on the fabric characteristics.

### 2.2. Characterization of Structure of Fabric Face Layer

**Figure 1:**
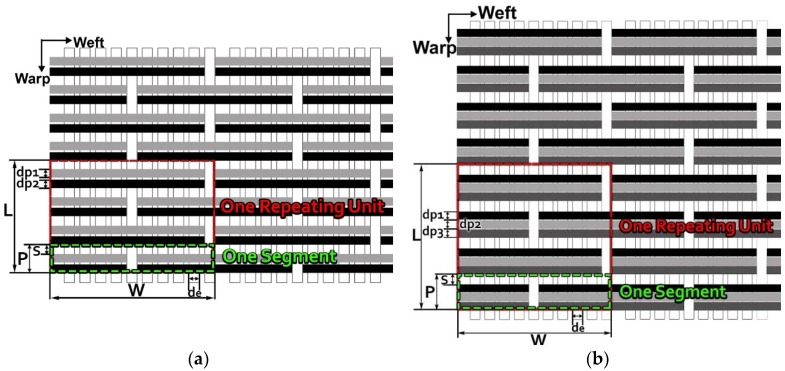
Diagrams of structures of fabric face layer: (**a**) RE-BLU mixing; (**b**) RE-BLU-BLK mixing.

The color attributes of woven fabric depend on not only the dyed-yarn components for mixing but also the arrangement of yarn floats in the fabric face layer. Therefore, in order to elucidate the relationship between the color attributes of fabric and the characteristics of structure of fabric face layer, some key geometrical parameters were identified to represent the structure of fabric face layer. Figure 1a,b display the diagrams of structures of the fabric by mixing blue and red color and blue, red, and black color on the fabric surface, respectively. In this study, one repeating unit is composed of ten warp yarns and twenty-eight or twenty-four weft yarns for the weft-backed structure with or without the regulating layer, respectively, wherein the face layer of fabric consists of eight wefts and twelve wefts for the blue-red and blue-red-black mixed fabrics, respectively. In terms of the arrangement of face weft yarns, the fabric face layer can be divided into four equal segments while each segment comprises of two and three face weft yarns for the blue-red and blue-red-black mixed fabrics, respectively. Considering the weave repeat unit, seven and eight key geometrical parameters, that is, labelled as parameters d_e_, d_p1_, d_p2_, d_p3_, W, L, P and S, were proposed for describing the geometry of fabric face layer for two and three dyed-yarn color mixing, respectively. d_e_ is the warp yarn diameter. d_p1_, d_p2_, and d_p3_ are the diameters of face differently dyed-weft yarns for mixing. W is the width of one repeating unit. L is the length of one repeating unit. P is the distance between two corresponding face-weft yarns in the two adjacent segments (length of segment), and S is the spacing between two adjacent face-weft yarns in different segments.

**Figure 2:**
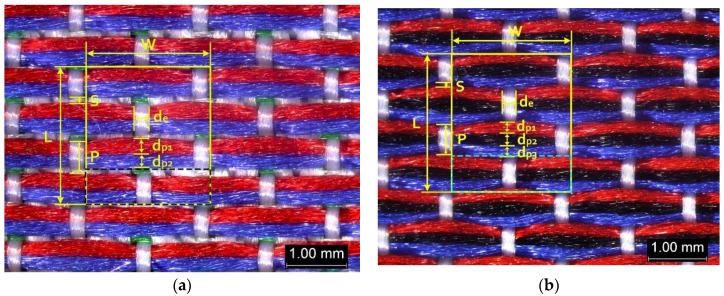
Application of characterization of geometry of fabric face layer: (**a**) RE-BLU mixing; (**b**) RE-BLU-BLK mixing.

Parameters d_e_, d_p1_, d_p2_, and d_p3_ represent the fineness of warps and face wefts. Parameters W and L describe the size of one repeating unit in terms of width and length. Parameter P represents the length of one segment, which is assumed to be equal to the ¼ length of the repeating unit. Parameter S focuses on the spacing between segments in the warp direction, which reflects the alignment of face weft yarns under a same binding unit by warps. It is assumed that all the weft yarns are in parallel position, and the diameter of the yarn with same yarn count should be uniform. The values of parameters W, L, and P are always positive theoretically while the value of the parameter S can be positive, zero, or negative. For the parameter S, a positive value represents that there is a spacing between two adjacent face weft yarns; zero means two adjacent face wefts yarn are aligned with each other without overlapping and spacing; and a negative value indicates that there are overlapping between two adjacent face weft yarns. To obtain the value of geometrical parameters from the produced woven fabric, pictures in microscopic view of the fabric were captured and scaled using the Leica M165 C Microscope (Leica Microsystems, Wetzlar, Germany); Figure 2 shows the application of this approach for characterizing the geometry of fabric face layer based on the microscopic images. The values of parameters were calculated and compared to investigate the effect of regulating layer, fabric density, weft yarn count, and fabric weave on the characteristics of structure of the fabric face layer, and thus, the resultant color attributes of fabrics.

### 2.3. Characterization of Fabric Physical Properties

By examining the relationship between fabric structural parameters and physical properties, the fabric parameters can be optimized to suit the requirement of different applications. The fabric thickness, fabric weight, and fabric tensile strength were measured and calculated to characterize the fabrics. For the fabric thickness, thickness gauge with 30.7 gf/cm^2^ weight was used following ASTM D1777-96. Three measurements were taken for each fabric sample in order to improve the accuracy and examine the variation of the measurement. According to ASTM D3776, an electronic balance was used to measure the weight of fabric samples. Three measurements were taken for each fabric sample. For the fabric tensile strength, Instron 4411 testing system (Instron, Norwood, MA, USA) with ASTM D5035 testing standard was used. The force required to break a specific width of fabric was determined by repeating the test three times in the warp and weft direction, respectively. By analyzing and comparing the data acquired from this section, the effects of regulating layer, fabric density, weft yarn count, and fabric weave on fabric physical properties were investigated.

### 2.4. Characterization of Fabric Color Attributes

To characterize the fabric color attributes, the color of the produced fabrics was measured and quantified using the 7000A spectrophotometer (X-Rite, Incorporated, MI, USA) [22,23]. The colorimetric data expressed by CIE L*, a*, b*, C*, H° values, which represents lightness, red-green component, blue-yellow component, chroma, and hue, respectively. In this study, the data was measured with the following specifications: small aperture, specular excluded, and UV excluded based on an illuminant D65 and 10° standard observer. All data acquired from this section were compared to investigate the effects of regulating layer, fabric density, weft yarn count, and fabric weave on the color attributes of fabrics.

## 3. Results and Discussion

### 3.1. Geometrical Parameters of Fabric Face Layer

#### 3.1.1. Influence of Weft-Backed Structure on Fabric Geometrical Parameters

**Figure 3:**
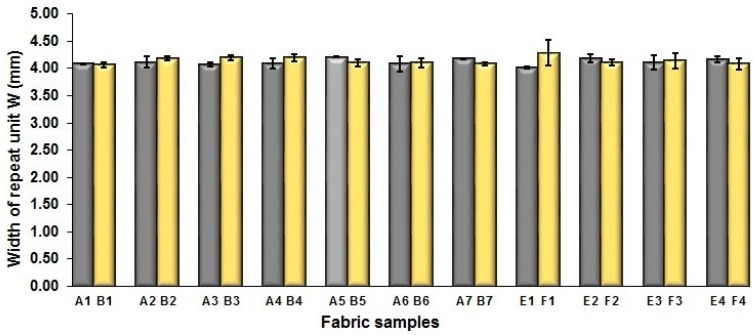
Effect of fabric weave on the width of repeating unit.

**Figure 4:**
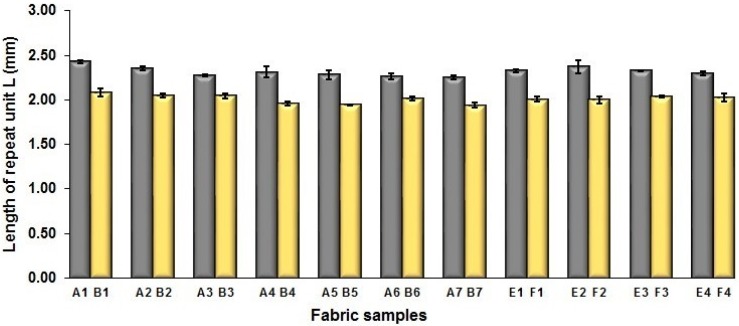
Effect of fabric weave on the length of one weave repeat.

To examine the effect of weft-backed structure with and without regulating layers on the fabric geometrical parameters, fabric samples A (1–7) and B (1–7) as well as E (1–4) and F (1–4) were taken into account. Figure 3 and Figure 4 show the effect of weft-backed structure on the width and the length of the weave repeating unit of the fabrics, respectively. Table 4 shows pictures of surfaces of some fabric samples. For the width shown in Figure 3, there are no significant differences between fabrics in two different structures wherein the average value obtained from BLU-RE mixing fabric is 4.12 mm and 4.13 mm for fabrics with and without middle layer, respectively, because these two types of fabrics have the same warp density. The average values of the repeat unit width for blue-red-black mixing fabrics with and without regulating layer are 4.12 mm and 4.16 mm, respectively. As shown in Figure 4, the fabric samples without the regulating layers B (1–7) and F (1–4) exhibit a lower length of weave repeat unit compared to that of fabrics with the regulating layers A (1–7) and E (1–4), respectively, in which the reduction of length from the fabric with regulating layer to fabric without regulating layer reaches 13.16% and 13.50% between the comparison pairs of B/A and F/E, respectively. This implies that weft yarns can be arranged more compactly in the fabrics without regulating layers compared to that of samples with regulating layers.

**Figure 5:**
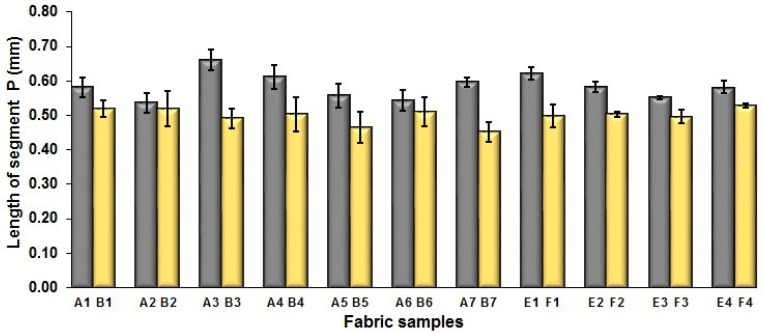
Effect of fabric weave on the length of one segment.

**Figure 6:**
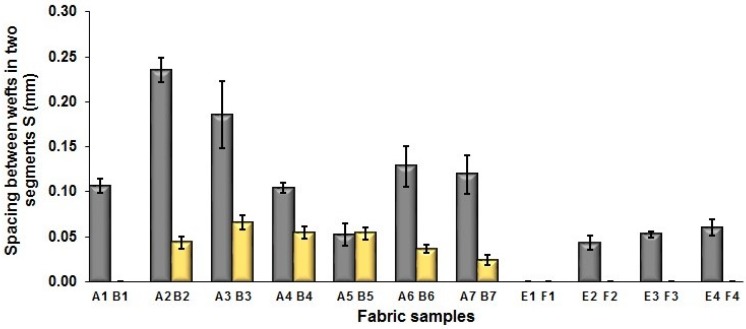
Effect of fabric weave on the distance between two adjacent face weft yarns.

In Figure 5, the same situation as that in Figure 4 is observed. The segment length of fabric sets A and E is larger than that of fabric sets B and F, respectively, with the average percentage decrease of 15.41% and 13.18% from the fabrics with regulating layer to fabrics without regulating layer for each comparison pair. Figure 6 shows the comparison of face weft yarn spacing S of each pair of two- and three-color mixed fabric samples. As shown in Figure 6, there is a significant difference between two types of weft-backed fabrics. This implies that by using same designed weft density and level of cramming motion, the face weft yarns of the fabric samples without the regulating layer are easy to approach together compared to the samples with the regulating layer due to the existing of the middle regulating layer in these fabrics, which results in the higher face weft density and cover factor in the fabrics without the middle regulating layer than that of fabrics with the regulating layer. Consequently, lower values of L and S in the fabric sets B and F can be achieved for better color effects.

#### 3.1.2. Influence of Fabric Density on Fabric Geometrical Parameters

**Figure 7:**
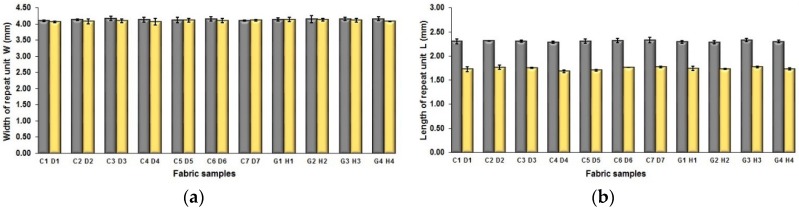

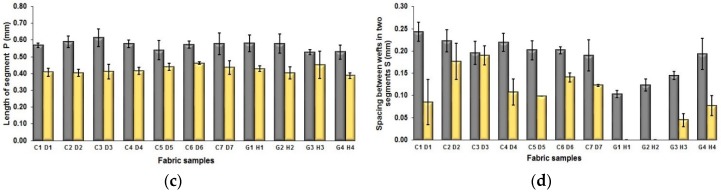
Effect of fabric density on the geometrical parameters: (**a**) width of repeating unit; (**b**) length of repeating unit; (**c**) length of segment; (**d**) spacing between two adjacent face weft yarns in different segments.

To investigate the effect of fabric density on fabric geometrical parameters, fabric samples C (1–7) and G (1–4) with a higher weft density of 160 picks/cm are compared to fabric samples D (1–7) and H (1–4) with a lower weft density of 120 picks/cm, respectively. Referring to Figure 7a,b, they show the comparison of width and length of the repeating unit between two sets of fabric samples. From Figure 7a, it can be observed that the variation is not significant, with only average difference of 0.039 mm between the comparison sample pair of C/D and average difference of 0.035 mm between the comparison pair of G/H. As shown in Figure 7b, the difference of the length of repeating unit is significant wherein the reduction of L reaches 32.76% and 24.14% for the comparison pairs C/D and G/H, respectively when a higher weft density is used for the fabrics. The effects of weft yarn density on the length of segment P and the spacing between two adjacent face wefts S are presented in Figure 7c,d, respectively. Similar to the effect on the length of repeat unit L, the values of P and S reduce significantly after the weft yarn density of fabric is increased from 120 picks/cm to 160 picks/cm. The decrease of L value is 35.45% and 24.37% for the comparison pair of fabrics C/D and G/H, respectively. In Figure 7d, the spacing between segments of fabric sample C (1–7) and G (1–4) shows a higher value than that of fabric samples D (1–7) and H (1–4) for the respective comparison pair of fabrics. The means of those samples were compared, and the results of Independent-Samples *t* test are presented in Table 7. The surface pictures of three pairs of samples (C5/D5, C6/D6, and G1/H1) are displayed in Table 8. The result matches with expectation since the fabric in a higher density means such fabric contains more yarns in the same unit length or area when comparing to the fabric with a lower density.

#### 3.1.3. Influence of Weft Yarn Linear Density on Fabric Geometrical Parameters

**Figure 8:**
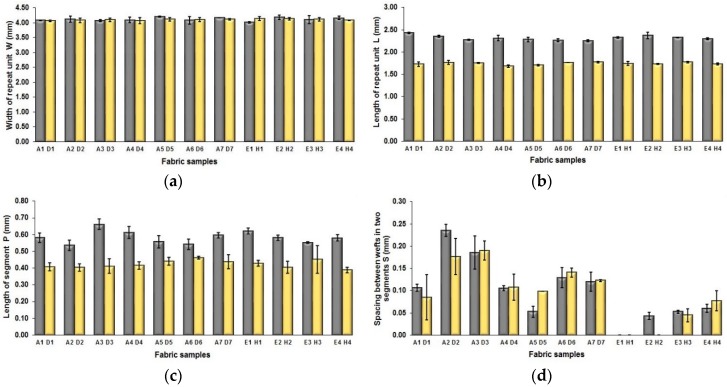
Effect of weft yarn count on the geometrical parameters: (**a**) width of repeating unit; (**b**) length of repeating unit; (**c**) length of one segment; (**d**) spacing between two adjacent face weft yarns in different segments.

The effect of weft yarn count on the fabric geometrical parameters is presented in Figure 8. Table 7 shows pictures of the surface structure of some fabric samples, including A5/D5, A6/D6, and E1/H1. As shown in Figure 8a, it is easy to see that the fabric samples A (1–7) and E (1–4) have similar width of repeat unit to that of fabric samples D (1–7) and H (1–4), respectively, because they have the same warp density. Figure 8b shows the comparison of length of weave repeat between each pair of fabric samples A/B and E/H. It can be seen that the difference is significant wherein the reduction of L from fabric samples A to D and E to H reach 32.76% and 25.12%, respectively. This can be ascribed to the use of finer weft yarns of 75 D in the fabric samples D (1–7) and H (1–4) compared to the samples A (1–7) and E (1–4) that used thicker weft yarns of 150 D.

The same situation is observed from Figure 8c, which shows the comparison of length of one segment (parameter P) between fabric samples using finer and thicker weft yarns. The average difference between two sets of fabric samples A/D is 0.158 mm wherein the decrease reaches 37.09%, while the difference between the comparison pair of E/H is 0.165 mm with the reduction of 28.25% from samples E to H. Figure 8d shows the difference between the comparison pairs of A/D and E/H in terms of the spacing between two adjacent face weft yarns in different segments. It can be seen that fabric samples D1, D2, H2, and H3 have smaller spacing than that of corresponding comparison samples A1, A2, E2, and E3. In addition, it is easy to observe that the spacing S of the blue-red-black samples E (1–4) and H (1–4) is much smaller compared to the fabric sample A (1–7) and D (1–7), which may indicate that the mixing of the component of black yarn leads to the decrease of the spacing between face yarn and thus lessen the exposing of white warp on the fabric face. The comparison of each pair of samples were conducted by using statistical analysis, and the results are listed in Table 9.

### 3.2. Fabric Physical Property

#### 3.2.1. Fabric Thickness

**Figure 9:**
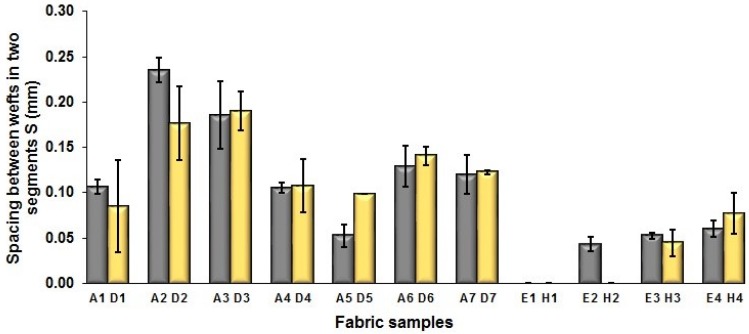
Comparison of fabric thickness.

The measured values of thickness of fabric samples are presented in Figure 9. For the comparison between different fabric sample sets, when comparing fabric sample set A and set E to set B and set F, respectively, the effect of weft-baked structure, with and without regulating layer, can be examined. It is seen that the fabric samples with the regulating layer possess a slightly higher thickness than that of fabrics without the regulating layer. To examine the effect of weft density on the fabric thickness, fabric sample sets C and G are compared to fabric sample sets D and H, respectively. Figure 9 illustrates that fabric sample sets D and H with a higher weft density are thicker than that of fabric sample sets C and G, respectively. Figure 9 also exhibits that the fabric sample sets A, E, B, and F have significantly higher fabric thickness than that of fabric sample sets C, G, D, and H, with the average percentage increase of 46.52%. It shows the effect of weft yarn count on the fabric thickness wherein the fabric sample sets A, E, B, and F were fabricated by using a higher weft yarn count (150 denier) while the fabric sample sets C, G, D, and H were produced with 75 denier weft yarns. Through the comparison of the fabric sample set A to D without the mixing component of black yarn with their corresponding fabric sample set E to H with the mixing component of black yarn shown in Figure 9, the effect of face structure, with or without the mixing component of black yarn, can be examined. It is easy to see that each comparison group does not show a significant difference, which indicates that the fabric thickness is not significantly affected when the black yarn is added into the fabric face layer for color mixing.

#### 3.2.2. Fabric Weight

**Figure 10:**
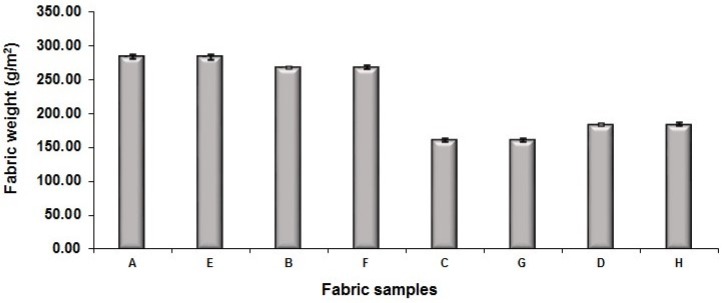
Comparison of fabric weight.

Figure 10 shows the comparison of fabric weight between eight sets of fabric samples. To investigate the effect of different factors on the fabric weight, the examination of different comparison pairs was conducted. Fabric set A and set E are compared to set B and set F, respectively, to evaluate the effect of weft-backed structure, with or without regulating layer, on the fabric weight. The weight of fabric set A and set E is heavier than that of fabric sets B and F, respectively, which indicates that the fabric weight is slightly increased by adding the regulating layer to the weft-backed structure. By comparing fabric sample sets C and G to fabric sample sets D and H, respectively, to examine the effect of weft density on the fabric weight, it can be seen that the fabric sample sets D and H in a higher weft density are heavier than that of the fabric sample sets C and G in a lower weft density, respectively. From Figure 10, fabric sample sets A, E, B, and F have significantly higher fabric weight than that of fabric sample sets C, G, D, and H, with the average percentage increase of 37.40% because a courser weft yarn of 150 denier was used in the fabric sample sets A, E, B, and F compared to the weft yarn in a count of 75 denier employed in the fabric sample sets C, G, D, and H. To investigate the effect of face structure with or without the mixing component of black yarn, the fabric sample set A to D without the mixing component of black yarn are compared to their corresponding fabric sample set E to H with the black yarn. It is easy to see that the adding of the black yarn into the fabric face layer shows no significant effect on the fabric weight. In addition, by comparing the result shown in Figure 10 to that in Figure 9, it is easy to observe that they have similar trends, which reflects that fabric thickness has a relationship with fabric weight.

#### 3.2.3. Fabric Tensile Strength

**Figure 11:**
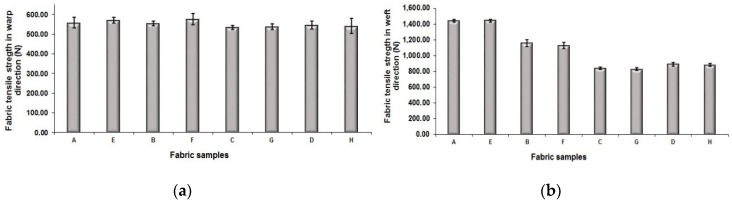
Comparison of fabric tensile property: (**a**) in warp-wise; (**b**) in weft-wise.

The measured values of fabric tensile strength in the warp and weft directions are presented in Figure 11a,b, respectively. As shown in Figure 11a, it can be seen that fabric samples set A and set E with the regulating layers possess a similar tensile strength in the warp direction to that of the fabric samples set B and set F without the regulating layer, respectively. It can be ascribed to the use of the same warp yarns and warp density in all eight fabric samples with and without the regulating layers. For the fabric tensile strength in the weft direction, the fabric samples with the regulating layers exhibit much higher tensile strength compared to that of fabrics without the regulating layers. The possible reason is that the integration of weft yarns in different layers is enhanced after adding the middle regulating layer for the fabric. To examine the effect of weft density on fabric tensile strength, fabric sample sets C and G are compared to fabric sample sets D and H. From the graph, it is seen that the fabric sample sets D and H with higher weft density are stronger than that of fabric sample set C and G. As shown in Figure 11b, fabric sample sets A, E, B, and F have significantly higher fabric tensile strength than that of fabric sample sets C, G, D, and H, respectively, with the average percentage increase of 33.45%. The main reason is that the weft yarns of 150 denier were used in the fabric sample sets A, E, B, and F while the weft yarn count of fabric sample sets C, G, D, and H is 75 denier. Lastly, to investigate the effect of face structure with or without the mixing component of black yarn on the fabric tensile strength, the fabric sample sets A, B, C, and D can be compared to their corresponding fabric sample sets E, F, G, and H, respectively. It can be observed that each comparison group does not show significant difference, which indicates that the introduction of black yarn for mixing into the fabric face layer does not have great influence on the fabric tensile strength.

### 3.3. Fabric Color Attributes

#### 3.3.1. Influence of Fabric Weave on Mixed Color Performance of Fabric

**Figure 12:**
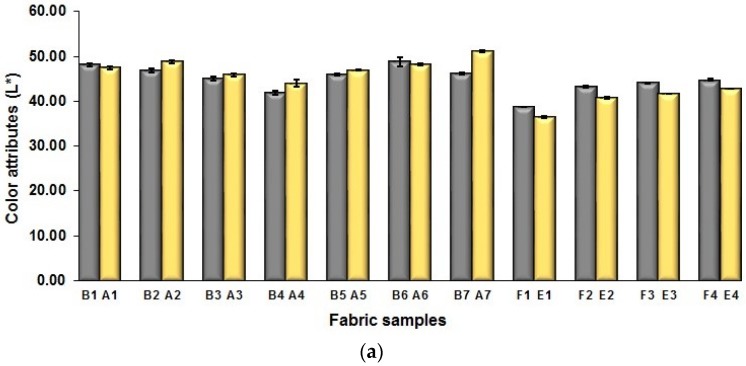

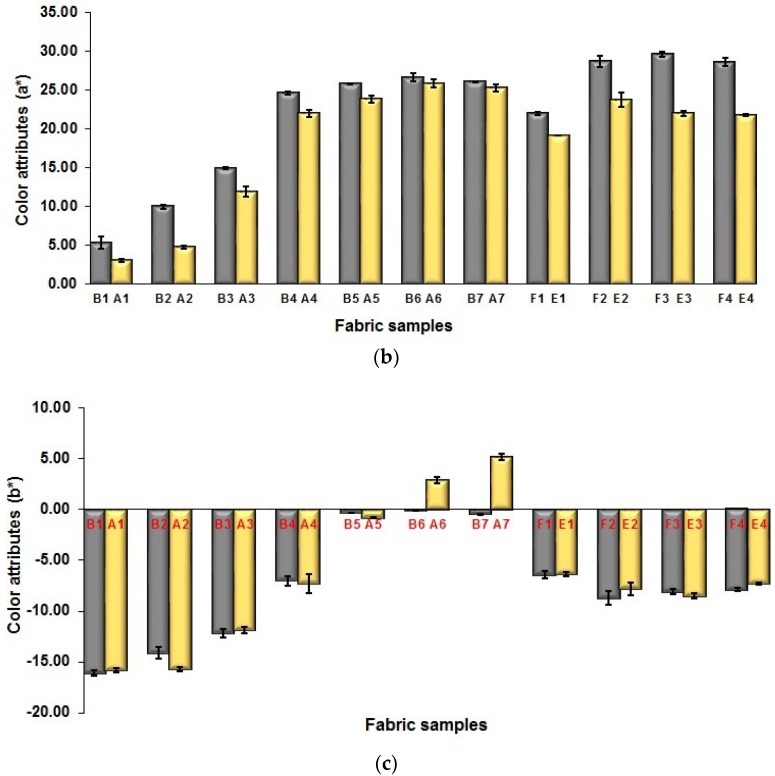
Comparison between fabrics in different fabric weave in terms of fabrics’ color performance: (**a**) L*; (**b**) a*; (**c**) b*.

Figure 12a shows the influence of fabric weave on the lightness (L*) of color mixed woven fabrics. As shown in Figure 12a, there is no significant difference in lightness between the fabrics with middle layers (A1 to A7) and their corresponding fabrics without middle layers (B1 to B7). As above-mentioned, for the fabrics (B1/A1 to B7/A7), the length of one repeating unit of fabric samples with regulating layers A (1–7) is larger than that of fabric samples without regulating layers B (1–7), and the space between weft yarns of fabrics with regulating layer is larger than that of fabrics without regulating layer, as shown in Figure 4 and Figure 6, which may result in the appearance of more white-warp floats on the fabric face and thus an increase of the fabric lightness. On the other side, more black-weft floats in the middle regulating layer are probably seen from the weft yarn space that leads to the decrease of fabric lightness. Moreover, the effect of structure on the fabric lightness most likely lowers due to the implementation of cramming motion. Figure 12b,c present the influence of fabric weave on the redness and blueness of fabrics. After adding a middle-regulating layer into the structure, the blue-red mixed fabrics with regulating layers (A1 to A7) have lower redness and varied blueness compared to the fabrics without regulating layers (B1 to B7). Again, the result is possibly caused by the application of cramming motion, which greatly increases the fabric tightness that makes the effect of regulating layer less obvious.

#### 3.3.2. Influence of Weft Density of Fabric on Mixed Color Attributes of Fabric

**Figure 13:**
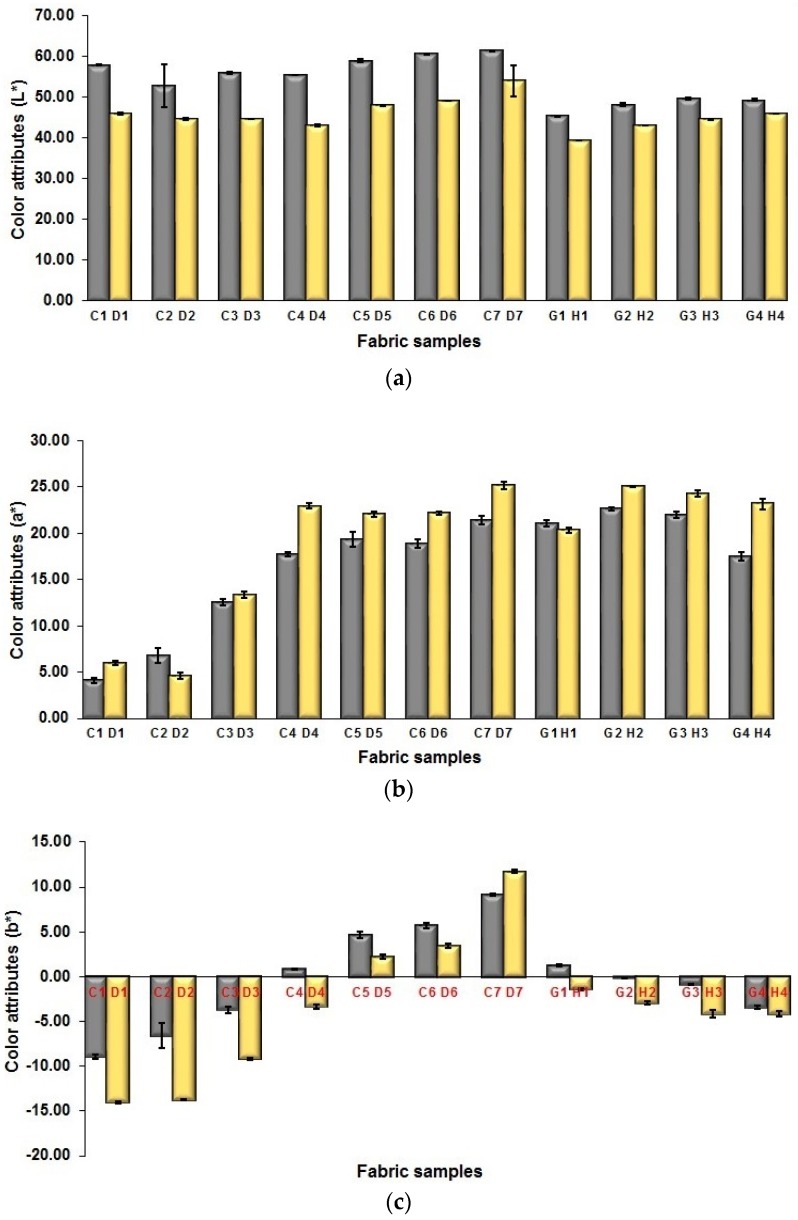
Comparison between fabrics in different weft density in terms of fabrics color performance: (**a**) L*; (**b**) a*; (**c)** b*.

Figure 13a shows the changes in lightness of blue-red mixing fabrics between fabric samples in a higher weft density and a lower weft density. Fabrics made by using lower weft yarn density have a higher lightness value than that of fabrics made by using higher weft yarn density. From the above-mentioned analysis of fabric geometrical parameters with higher and lower densities, the length of one repeating unit of the higher density fabric is shorter than that of the higher density fabric, which indicates that the weft yarns in the fabric with a higher fabric density align closer than that of the corresponding fabric sample with a lower fabric density. The weft yarn spacing between two adjacent segments of high density fabrics is smaller than that of lower density fabrics. Consequently, there are less white warp yarns exposed in the higher density fabrics surface than that in the lower density fabrics, which results in the lowering of fabric lightness through increasing the fabric weft density. Figure 13b,c explain the dependence of fabric redness-greenness and blueness-yellowness on the weft yarn density, respectively. It can be seen that the colored yarn mixed fabrics in a higher density have higher redness (a*) and blueness (b*) than those in a lower density. As mentioned above, the space between weft yarns of higher density fabrics is smaller than that of lower density fabrics, and thus, the face weft yarn packed more closely for the high-density fabric. Table 10 shows the results of Independent-Samples *t* test on each pair of samples in terms of color performance.

#### 3.3.3. Influence of Weft Count of Fabric on Mixed Color Attributes of Fabric

**Figure 14:**
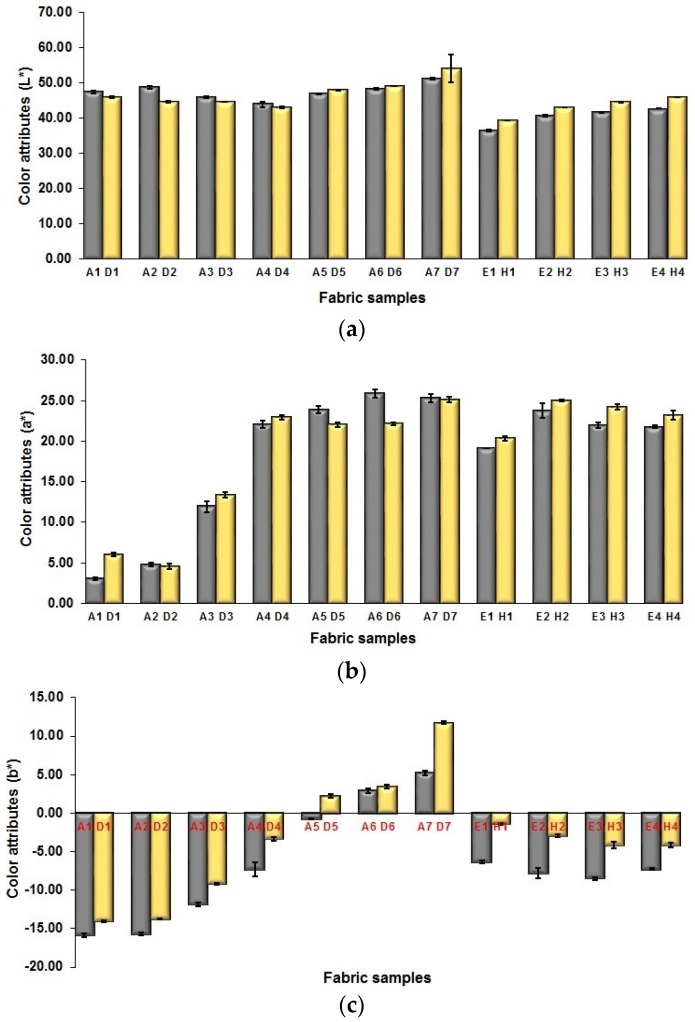
Comparison between fabrics in different weft linear density in terms of fabrics’ color performance: (**a**) L*; (**b**) a*; (**c**) b*.

The lightness of blue-red mixing fabrics by using thicker or finer weft yarns are shown in Figure 14a. As shown in Figure 14a, it is easy to see that the fabric samples D (1–7) with a finer weft yarn of 75 denier exhibit similar or lower fabric lightness compared to their corresponding fabric samples A (1–7) using a thicker weft yarn of 150 denier. Based on the above-mentioned geometrical parameter values of fabric face layer shown in Figure 8, the relative closeness or cover factor of face weft yarns in the fabric samples D (1–7) using the finer weft yarns is lower than that of fabric samples A (1–7) using the thicker weft yarns by employing cramming motion during the weaving. However, when comparing with the thicker weft yarn fabrics, the finer weft yarn fabrics still can achieve the similar or even lower lightness. Figure 14a also displays the comparison of lightness of each pair of BLU-RE-BLK mixed fabrics. It can be seen that the finer weft yarn fabric samples have higher lightness than that of fabric samples using the thickness yarns. Figure 14b,c present the comparison of fabrics using thicker and finer weft yarns in terms of the redness and greenness (a*) and blueness and yellowness (b*). As shown in Figure 14b,c, the majority of fabric samples produced by using finer weft yarns have higher redness than that produced by using thicker weft yarns, while the blueness of finer weft yarn fabrics is lower compared to their corresponding thicker weft yarn fabric samples.

#### 3.3.4. Influence of Mixing Component of Black Yarn on Fabric Color Attributes

**Figure 15:**
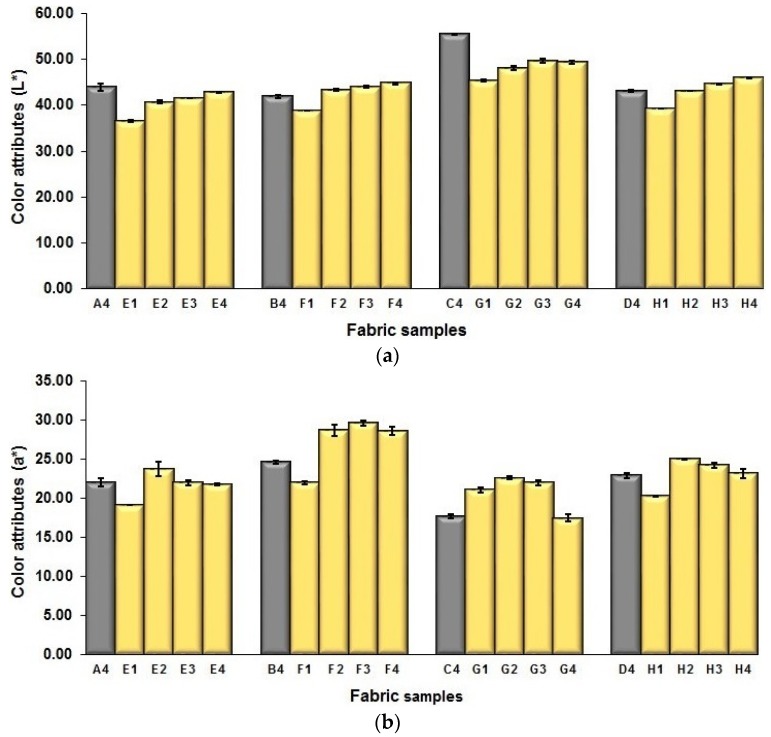

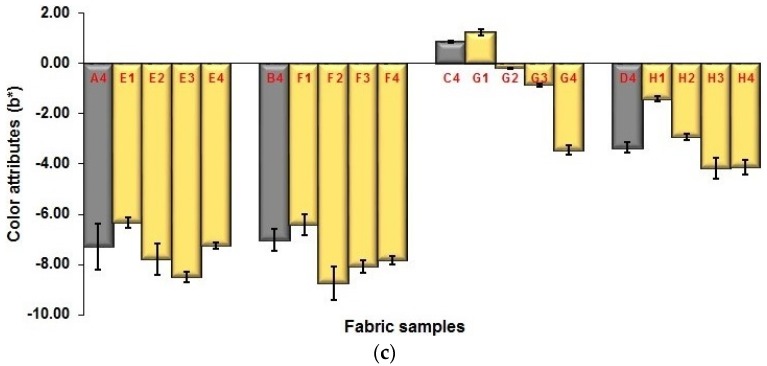
Influence of adding black yarn floats on the fabric color performance: (**a**) L*; (**b**) a*; (**c**) b*.

To lower the lightness of fabrics due to the white warp yarns used, black weft yarns were added into the face layer of fabric for mixing with the blue and red weft yarns of the above-mentioned blue-red mixed fabrics. Based on the weaves M_(BL+R)_ (4) and N_(BL+R)_ (4) for the blue-red mixed fabrics, new weaves M_(BL+R+BK)_ (1–4) and N_(BL+R+BK)_ (1–4) with black weft floats in diverse ratios were designed for adding the black yarn floats into the blue–red mixed fabrics with and without middle layers, respectively. Figure 15a presents the effect of mixing proportions of black yarn on the lightness of blue–red–black mixed fabrics wherein A4, B4, C4, and D4 are blue–red mixed fabrics, and E(1–4), F(1–4), G(1–4), and H(1–4) are their corresponding blue–red–black mixed fabrics in different black mixing proportion. As shown in Figure 15a, with the black yarns added to the surface of fabrics, the lightness of fabrics with and without the regulating layers, in lower and higher weft density or by using the thicker and finer yarns, shows a similar trend, in which there is significant difference between each pair of samples A4 and E1, B4 and F1, C4 and G1, as well as D4 and H1. For each set of E, F, G, and H fabrics with black yarns within the face layer of the fabric, E1, F1, G1, and H1 have the highest proportion of black components, and thus, the reduction of fabric lightness to the fabrics without the black yarns is the biggest. Figure15b,c present the change of color performance (a* and b*) by adding black yarn floats in varied proportion into the fabric surface. As shown in Figure 15b,c, the redness-greenness and blueness-yellowness varied with the introduction and increase of the proportion of black yarn floats on the fabric face layer.

### 3.4. Comparison of Lightness of Fabrics Produced with and without Cramming Motion

**Figure 16:**
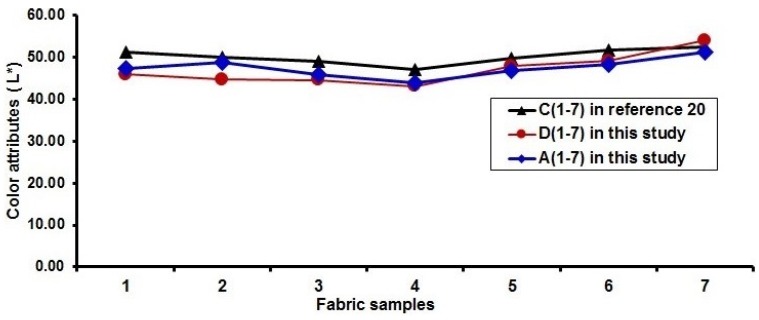
Comparison of the fabric lightness with blue-red fabric without cramming motion [20].

**Figure 17:**
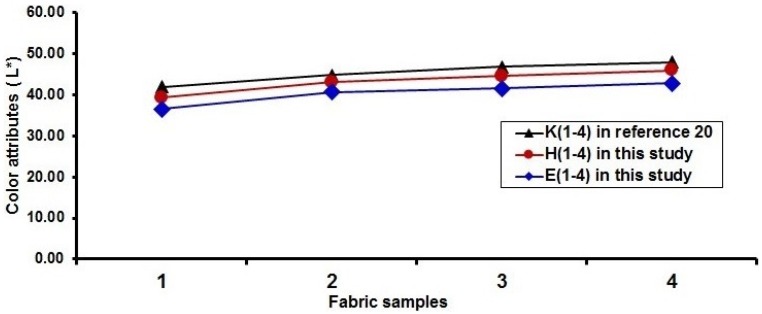
Comparison of the fabric lightness with blue-red-black fabric without cramming motion [20].

As mentioned above, in order to lower the lightness of fabric surface due to the white warps used, cramming motion and finer yarns were used for fabricating the blue-red and blue-red-black mixed fabrics. Figure 16 shows a comparison of the lightness of blue-red mixed fabrics by using cramming motion and finer yarn in the present study with that without cramming motion in the previous study [20]. It can be seen that, for each pair of blue-red mixed fabrics without and with cramming motion, the fabrics with cramming motion and finer yarns (D1 to D7 and A1 to A7 in this study) possess lower values in lightness compared to their corresponding fabrics without cramming motion (C1 to C7 in Reference [20]), except the sample D7. In the case of a pair of fabrics (D2/C2), the fabric sample D2 using cramming motion and finer yarns has a lightness reduction of around 11% for 50 to 44. The effect of using cramming motion on the lightness of blue-red-black mixed fabrics is presented in Figure 17. The comparison shows a similar trend in terms of fabric lightness. As shown in Figure 17, the fabric sample E1 can achieve the fabric lightness reduction of about 13% by using the cramming motion compared to the fabric sample K1 without cramming motion. The possible reason is that the weft yarn density and cover factor of fabric face layer are increased by applying cramming motion and finer yarns for fabricating the blue-red and/or black mixed fabrics. In this study, the weft yarn ratio of face to back in one segment for the weft-backed woven structure without the regulating layer is 2 to 4, and the weft-backed fabric with the regulating layer has the weft yarn ratio of face to middle to back in one segment of 2 to 1 to 4. This means that the back layer of fabric has more weft yarns arranged parallel than that of the face layer of fabric. Therefore, the possible overlap of back weft yarns due to the cramming motion applied may lead to the close arrangement of the face weft yarns of the fabric. This demonstrates that employing the cramming motion and finer wefts for the production of dyed-weft yarn centered colorful fabrics is an effective way to enhance the color attributes of fabrics.

## 4. Conclusions

In this paper, the color mixed woven fabrics with enhanced color attributes can be obtained from interlacing the raw-white warp yarns with the multi-colored weft yarns through the implementation of cramming motion during the weaving and using finer weft yarns. The result reflects that the fabric structure and density, weft yarn count, and the introduction of black yarn on the fabric face layer have effects on the geometrical parameters of fabric surface, and thus, the final color attributes of fabrics. Considering the weave repeat unit, key geometrical parameters were proposed for describing the geometry of fabric face layer for dyed-yarn color mixing. The experimental results show the fabric samples without the regulating layers exhibit a lower length of weave repeat unit compared to that of fabrics with the regulating layers. The length of segment and spacing between weft yarns reduce significantly after the weft yarn density of fabric increases. The results indicate that the mixing of the component of black yarn leads to the decrease of the spacing between face yarn and thus lessens the exposing of white warp on the fabric face. In terms of fabric physical properties, the results show that the fabric samples with the regulating layer possess a slightly higher thickness and much higher tensile strength in the weft direction than that of fabrics without the regulating layer due to the enhanced fabric structure with the regulating layer. By employing the cramming motion, the weft density and cover factor of fabric face layer are increased, which results in the lowering of fabric lightness and enhancement of redness and blueness through increasing the fabric weft density. When comparing with the thicker weft yarn fabrics, the finer weft yarn fabrics still can achieve the similar or even lower lightness under the condition of slightly lower weft yarn cover factor of fabric. The comparison of fabrics’ color attributes between samples, with and without the applying of cramming motion, demonstrates that employing the cramming motion and finer wefts for the production of dyed-weft yarn-centered colorful fabrics is an effective way to enhance the color attributes of fabrics.

## Figures and Tables

**Table 1:** Fabric specifications.

Fabric Sample	Yarn Count (Denier)		Yarn Color		Weave Code	Fabric Density (Threads/cm)	
	Warp	Weft	Warp	Weft Yarn Color Mixing		Warp	Weft
A (1–7)	100	150	White	Blue + Red	M_(BL+R)_ (1–7)	47	120
B (1–7)	100	150	White	Blue + Red	N_(BL+R)_ (1–7)	47	120
C (1–7)	100	75	White	Blue + Red	M_(BL+R)_ (1–7)	47	120
D (1–7)	100	75	White	Blue + Red	M_(BL+R)_ (1–7)	47	160
E (1–4)	100	150	White	Blue + Red + Black	M_(BL+R+BK)_ (1–4)	47	120
F (1–4)	100	150	White	Blue + Red + Black	N_(BL+R+BK)_ (1–4)	47	120
G (1–4)	100	75	White	Blue + Red + Black	M_(BL+R+BK)_ (1–4)	47	120
H (1–4)	100	75	White	Blue + Red + Black	M_(BL+R+BK)_ (1–4)	47	160

**Table 2:** Yarn color performance.

Color Attributes	Weft Thread						Warp Thread
	RE	GR	BLU	YE	BLK	Wp	We
**Yarn Sample**	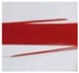	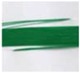	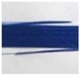	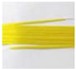	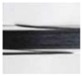	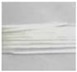	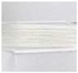
L*	44.87	51.01	29.02	89.75	17.41	92.93	91.00
a*	56.38	−50.80	10.55	−12.06	−0.92	4.30	0.24
b*	42.70	31.47	−39.65	88.63	−1.26	−14.94	0.87
C*	70.73	59.76	41.03	89.45	1.56	15.55	0.90
h°	37.14	148.22	284.90	97.75	306.14	286.06	285.42

**Table 3:** Fabric weaves designed for BLU and RE wefts mixing on the fabric surface (M represents the weave with regulating layer).

Weave Code	M_(BL+R)_ (1)	M_(BL+R)_ (2)	M_(BL+R)_ (3)	M_(BL+R)_ (4)	M_(BL+R)_ (5)	M_(BL+R)_ (6)	M_(BL+R)_ (7)
Face weave	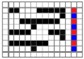	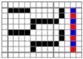	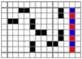	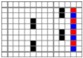	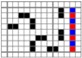	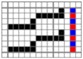	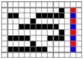
Middle regulating weave	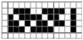	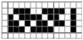	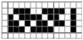	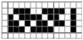	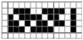	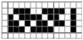	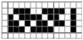
Back weave	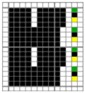	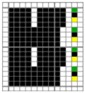	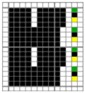	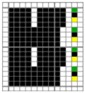	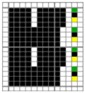	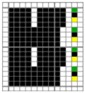	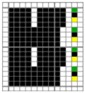

**Table 4:** Fabric weaves designed for BLU, RE, and BLK wefts mixing on the fabric surface (M represents the weave with regulating layer).

Weave Code	M_(BL+R+BK)_ (1)	M_(BL+R+BK)_ (2)	M_(BL+R+BK)_ (3)	M_(BL+R+BK)_ (4)
Face weave	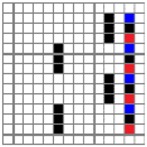	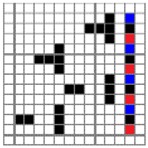	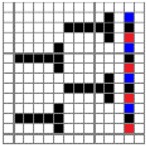	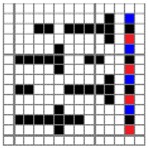
Middle regulating weave	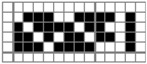	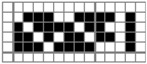	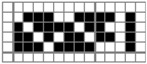	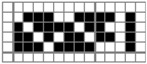
Back weave	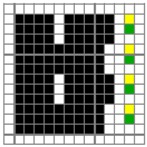	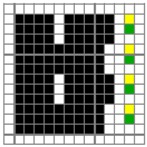	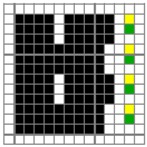	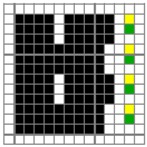

**Table 5:** Fabric weaves designed for BLU and RE wefts mixing on the fabric surface (N represents the weave without regulating layer).

Weave Code	N_(BL+R)_ (1)	N_(BL+R)_ (2)	N_(BL+R)_ (3)	N_(BL+R)_ (4)	N_(BL+R)_ (5)	N_(BL+R)_ (6)	N_(BL+R)_ (7)
Face weave	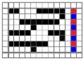	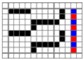	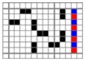	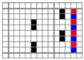	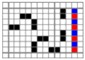	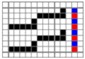	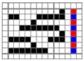
Back weave	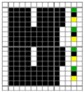	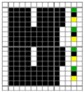	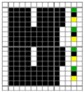	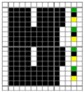	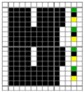	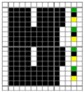	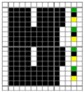

**Table 6:** Fabric weaves designed for BLU, RE, and BLK wefts mixing on the fabric surface (N represents the weave without regulating layer).

Weave Code	N_(BL+R+BK)_ (1)	N_(BL+R+BK)_ (2)	N_(BL+R+BK)_ (3)	N_(BL+R+BK)_ (4)
Face weave	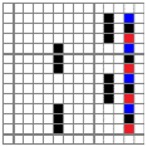	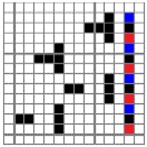	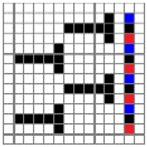	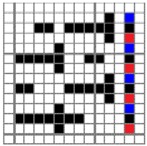
Back weave	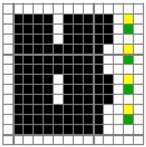	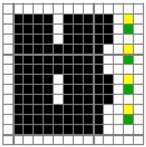	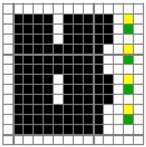	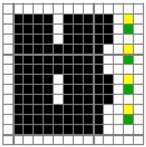

**Table 7:** Results of Independent-Samples *t* test.

Fabric Sample No.	W		L		P		S	
	Mean Difference	Sig.*	Mean Difference	Sig.*	Mean Difference	Sig.*	Mean Difference	Sig.*
**C_1_D_1_**	0.037	0.094	0.573	0.000	0.158	0.001	0.159	0.005
**C_2_D_2_**	0.050	0.282	0.553	0.000	0.185	0.010	0.045	0.135
**C_3_D_3_**	0.787	0.183	0.549	0.000	0.202	0.004	0.007	0.703
**C_4_D_4_**	0.667	0.338	0.602	0.000	0.161	0.000	0.112	0.003
**C_5_D_5_**	0.003	0.954	0.603	0.000	0.987	0.036	0.103	0.001
**C_6_D_6_**	0.063	0.270	0.548	0.000	0.111	0.001	0.061	0.001
**C_7_D_7_**	−0.021	0.239	0.561	0.000	0.140	0.024	0.067	0.021
**G_1_H_1_**	0.002	0.969	0.548	0.000	0.152	0.005	0.103	0.000
**G_2_H_2_**	0.193	0.751	0.556	0.000	0.173	0.007	0.124	0.000
**G_3_H_3_**	0.036	0.413	0.556	0.000	0.074	0.156	0.100	0.000
**G_4_H_4_**	0.083	0.065	0.565	0.000	0.140	0.004	0.117	0.005

* The mean difference is significant at the 0.05 level.

**Table 8:** Microscopic view of surface of fabric samples.

**Fabric Name**	**B5**	**A5**	**D5**	**C5**
Microscopic view	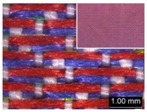	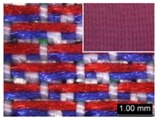	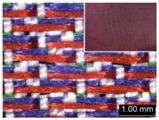	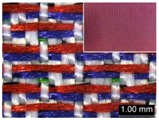
**Fabric Name**	**B6**	**A6**	**D6**	**C6**
Microscopic view	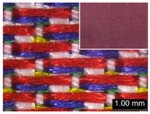	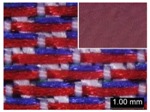	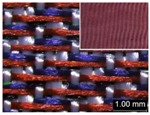	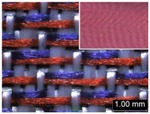
**Fabric Name**	**F1**	**E1**	**H1**	**G1**
Microscopic view	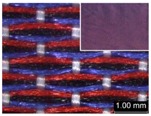	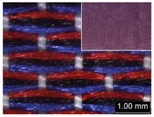	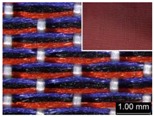	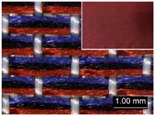

**Table 9:** Results of Independent-Samples *t* test.

Fabric Sample No.	W		L		P		S	
	Mean Difference	Sig.	Mean Difference	Sig.	Mean Difference	Sig.	Mean Difference	Sig.
**A_1_D_1_**	0.021	0.184	0.697	0.000	0.170	0.001	0.022	0.446
**A_2_D_2_**	0.037	0.592	0.589	0.000	0.132	0.002	0.058	0.056
**A_3_D_3_**	−0.021	0.601	0.520	0.000	0.249	0.001	−0.004	0.864
**A_4_D_4_**	0.025	0.737	0.630	0.000	0.195	0.001	−0.004	0.820
**A_5_D_5_**	0.096	0.044	0.576	0.000	0.116	0.005	−0.046	0.002
**A_6_D_6_**	−0.017	0.834	0.493	0.000	0.081	0.008	−0.012	0.390
**A_7_D_7_**	0.055	0.013	0.481	0.000	0.158	0.002	−0.003	0.776
**E_1_H_1_**	−0.119	0.027	0.580	0.000	0.193	0.000	0.000	0.000
**E_2_H_2_**	0.057	0.243	0.643	0.000	0.177	0.001	0.004	0.000
**E_3_H_3_**	−0.012	0.884	0.555	0.000	0.099	0.077	0.008	0.342
**E_4_H_4_**	0.079	0.047	0.564	0.000	0.194	0.000	−0.017	0.246

**Table 10:** Results of Independent-Samples *t* test.

Fabric Sample No.	L*		a*		b*	
	Mean Difference	Sig.	Mean Difference	Sig.	Mean Difference	Sig.
**C_1_D_1_**	11.912	0.000	−1.933	0.001	5.095	0.000
**C_2_D_2_**	8.148	0.041	2.229	0.007	7.179	0.001
**C_3_D_3_**	11.353	0.000	−0.821	0.030	5.461	0.000
**C_4_D_4_**	12.314	0.000	−5.215	0.000	4.216	0.000
**C_5_D_5_**	10.966	0.000	−2.690	0.003	2.455	0.000
**C_6_D_6_**	11.615	0.000	−3.278	0.000	2.284	0.000
**C_7_D_7_**	7.325	0.021	−3.687	0.000	−2.565	0.000
**G_1_H_1_**	6.100	0.000	0.751	0.027	2.654	0.000
**G_2_H_2_**	4.997	0.000	−2.396	0.000	2.739	0.000
**G_3_H_3_**	5.085	0.000	−2.228	0.001	3.317	0.000
**G_4_H_4_**	3.461	0.000	−5.679	0.000	0.694	0.025
